# Supplementary material for: Mobilization of LINE-1 retrotransposons is restricted by Tex19.1 in mouse embryonic stem cells
Source: eLife. 2017 Aug 14;6:e26152. doi: 10.7554/eLife.26152 (PMC5570191; doi:10.7554/eLife.26152)
Supplement: Supplementary file 1. — Preliminary mass spectrometry data obtained from a single immunoprecipitation and mass spectrometry experiment from ESC cytoplasmic lysates. TEX19.1-YFP and YFP alone immunoprecipitations were run on a polyacrylamide gel and each gel lane cut into seven bands according to size (T1-T7). Proteins identified by mass spectrometry in TEX19.1-YFP immunoprecipitates, but not in YFP controls, are listed in the table. This preliminary list could contain proteins that interact non-specifically with the beads or YFP that are variably detected in YFP alone samples, and/or proteins that are variably present in TEX19.1-YFP samples that cannot be consistently reproduced under these experimental conditions. Proteins that were verified as TEX19.1 interactors in independent samples by Western blotting are listed in Supplementary file 2. DOI: http://dx.doi.org/10.7554/eLife.26152.022 [file elife-26152-supp1.doc]

### Supplementary file 1. Preliminary Mass Spectrometry Data From TEX19.1-YFP Immunoprecipitates.

| **Band** | **Accession** | **Description** |
| --- | --- | --- |
| T1 | gi|27434482 | ubiquitin ligase E3 alpha-II [Mus musculus] |
| T1 | gi|60549637 | LASU1 [Mus musculus] |
| T1 | gi|74201741 | unnamed protein product [Mus musculus] |
| T1 | gi|6505713 | pericentriolar material-1 [Mus musculus] |
| T1 | gi|38503465 | keratin 17n [Mus musculus] |
| T1 | gi|237820660 | E3 ubiquitin-protein ligase UBR4 [Mus musculus] |
| T1 | gi|28077023 | testis-expressed protein 19.1 [Mus musculus] |
| T1 | gi|191765 | alpha-fetoprotein [Mus musculus] |
| T1 | gi|117938332 | spectrin beta chain, brain 1 isoform 1 [Mus musculus] |
| T1 | gi|200009 | myosin I [Mus musculus] |
| T1 | gi|115496850 | spectrin alpha chain, brain isoform 1 [Mus musculus] |
| T1 | gi|467517 | collagen [Mus musculus] |
| T1 | gi|123229143 | midasin homolog (yeast) [Mus musculus] |
| T1 | gi|9717245 | cytoplasmic dynein heavy chain [Mus musculus] |
| T1 | gi|12853209 | unnamed protein product [Mus musculus] |
| T1 | gi|47523977 | keratin, type II cytoskeletal 72 [Mus musculus] |
| T1 | gi|1293893 | leucine zipper protein 1 [Mus musculus] |
| T1 | gi|3642669 | ribosomal protein L3 [Mus musculus] |
| T1 | gi|26328229 | unnamed protein product [Mus musculus] |
| T1 | gi|47847514 | mFLJ00343 protein [Mus musculus] |
| T2 | gi|27434482 | ubiquitin ligase E3 alpha-II [Mus musculus] |
| T2 | gi|74184606 | unnamed protein product [Mus musculus] |
| T2 | gi|74184809 | unnamed protein product [Mus musculus] |
| T2 | gi|20070691 | Myh11 protein [Mus musculus] |
| T2 | gi|37360290 | mKIAA1227 protein [Mus musculus] |
| T2 | gi|13431704 | RecName: Full=Myosin-If |
| T2 | gi|3378046 | brush border myosin-I [Mus musculus] |
| T2 | gi|59798479 | RecName: Full=Keratin, type II cytoskeletal 6B |
| T2 | gi|1167510 | TI-225 [Mus musculus] |
| T2 | gi|28077023 | testis-expressed protein 19.1 [Mus musculus] |
| T2 | gi|74216080 | unnamed protein product [Mus musculus] |
| T2 | gi|124486959 | myosin-13 [Mus musculus] |
| T2 | gi|38173965 | Myh6 protein [Mus musculus] |
| T2 | gi|18859641 | myosin-7 [Mus musculus] |
| T2 | gi|32526605 | pol [Mus musculus] |
| T2 | gi|148684097 | mCG13235 [Mus musculus] |
| T2 | gi|1293893 | leucine zipper protein 1 [Mus musculus] |
| T2 | gi|111120334 | myosin-IIIb [Mus musculus] |
| T2 | gi|56206043 | kinesin family member 1C [Mus musculus] |
| T2 | gi|407339 | Kif1b [Mus musculus] |
| T3 | gi|27434482 | ubiquitin ligase E3 alpha-II [Mus musculus] |
| T3 | gi|1621617 | KRAB-A interacting protein [Mus musculus] |
| T3 | gi|28077023 | testis-expressed protein 19.1 [Mus musculus] |
| T3 | gi|309319 | heat shock protein 70 cognate [Mus musculus] |
| T3 | gi|37360290 | mKIAA1227 protein [Mus musculus] |
| T3 | gi|1304157 | 78 kDa glucose-regulated protein [Mus musculus] |
| T3 | gi|74143673 | unnamed protein product [Mus musculus] |
| T3 | gi|7638398 | epidermal keratin 10 [Mus musculus] |
| T3 | gi|1167510 | TI-225 [Mus musculus] |
| T3 | gi|111185567 | Krt78 protein [Mus musculus] |
| T3 | gi|148684097 | mCG13235 [Mus musculus] |
| T3 | gi|435838 | mortalin mot-1=hsp70 homolog cytosolic form [mice, CD1-ICR embryonic fibroblasts, MEF, Peptide, 679 aa] |
| T3 | gi|74201741 | unnamed protein product [Mus musculus] |
| T3 | gi|26328849 | unnamed protein product [Mus musculus] |
| T3 | gi|59798479 | RecName: Full=Keratin, type II cytoskeletal 6B |
| T3 | gi|26325114 | unnamed protein product [Mus musculus] |
| T3 | gi|6671684 | catenin beta-1 [Mus musculus] |
| T4 | gi|309319 | heat shock protein 70 cognate [Mus musculus] |
| T4 | gi|28077023 | testis-expressed protein 19.1 [Mus musculus] |
| T4 | gi|27434482 | ubiquitin ligase E3 alpha-II [Mus musculus] |
| T4 | gi|12963615 | tubulin beta-3 chain [Mus musculus] |
| T4 | gi|21746161 | tubulin beta-2B chain [Mus musculus] |
| T4 | gi|27754056 | tubulin beta-6 chain [Mus musculus] |
| T4 | gi|74181454 | unnamed protein product [Mus musculus] |
| T4 | gi|7638398 | epidermal keratin 10 [Mus musculus] |
| T4 | gi|309215 | EndoA' cytokeratin (5' end put.); putative [Mus musculus] |
| T4 | gi|26344812 | unnamed protein product [Mus musculus] |
| T4 | gi|14198122 | Grwd1 protein [Mus musculus] |
| T4 | gi|59798479 | RecName: Full=Keratin, type II cytoskeletal 6B |
| T4 | gi|74143673 | unnamed protein product [Mus musculus] |
| T4 | gi|74182195 | unnamed protein product [Mus musculus] |
| T4 | gi|12834430 | unnamed protein product [Mus musculus] |
| T4 | gi|460317 | chaperonin [Mus musculus] |
| T4 | gi|12805509 | Nop56 protein [Mus musculus] |
| T4 | gi|201725 | t complex polypeptide 1 [Mus musculus] |
| T4 | gi|74198645 | unnamed protein product [Mus musculus] |
| T4 | gi|12846632 | unnamed protein product [Mus musculus] |
| T4 | gi|6671531 | nuclear receptor subfamily 0 group B member 1 [Mus musculus] |
| T4 | gi|47523977 | keratin, type II cytoskeletal 72 [Mus musculus] |
| T4 | gi|435838 | mortalin mot-1=hsp70 homolog cytosolic form [mice, CD1-ICR embryonic fibroblasts, MEF, Peptide, 679 aa] |
| T4 | gi|347839 | matricin [Mus musculus] |
| T4 | gi|6755382 | ruvB-like 2 [Mus musculus] |
| T4 | gi|1167510 | TI-225 [Mus musculus] |
| T4 | gi|7305635 | ATP-dependent zinc metalloprotease YME1L1 [Mus musculus] |
| T5 | gi|4501887 | actin, cytoplasmic 2 [Homo sapiens] |
| T5 | gi|74213524 | unnamed protein product [Mus musculus] |
| T5 | gi|74204169 | unnamed protein product [Mus musculus] |
| T5 | gi|387090 | alpha-cardiac actin [Mus musculus] |
| T5 | gi|148698861 | prolyl-tRNA synthetase (mitochondrial)(putative), isoform CRA_a [Mus musculus] |
| T5 | gi|27434482 | ubiquitin ligase E3 alpha-II [Mus musculus] |
| T5 | gi|34740335 | tubulin alpha-1B chain [Mus musculus] |
| T5 | gi|12963615 | tubulin beta-3 chain [Mus musculus] |
| T5 | gi|74191638 | unnamed protein product [Mus musculus] |
| T5 | gi|38503465 | keratin 17n [Mus musculus] |
| T5 | gi|28077023 | testis-expressed protein 19.1 [Mus musculus] |
| T5 | gi|12853521 | unnamed protein product [Mus musculus] |
| T5 | gi|387398 | epidermal keratin type I [Mus musculus] |
| T5 | gi|254281204 | L-threonine 3-dehydrogenase, mitochondrial precursor [Mus musculus] |
| T5 | gi|19527048 | heterogeneous nuclear ribonucleoprotein F [Mus musculus] |
| T5 | gi|148692199 | translocase of inner mitochondrial membrane 50 homolog (yeast), isoform CRA_a [Mus musculus] |
| T5 | gi|110625979 | elongation factor 1-gamma [Mus musculus] |
| T5 | gi|9789937 | dnaJ homolog subfamily A member 2 [Mus musculus] |
| T5 | gi|6671539 | fructose-bisphosphate aldolase A isoform 2 [Mus musculus] |
| T5 | gi|19527242 | tubulin gamma-1 chain [Mus musculus] |
| T5 | gi|1167510 | TI-225 [Mus musculus] |
| T5 | gi|532211 | Y-box binding protein [Mus musculus] |
| T5 | gi|21432060 | Wdr18 protein [Mus musculus] |
| T5 | gi|6680297 | dnaJ homolog subfamily A member 1 [Mus musculus] |
| T5 | gi|53543 | Otx2 [Mus musculus] |
| T5 | gi|10946972 | coatomer subunit epsilon [Mus musculus] |
| T5 | gi|12839434 | unnamed protein product [Mus musculus] |
| T5 | gi|309319 | heat shock protein 70 cognate [Mus musculus] |
| T5 | gi|263310 | transforming growth factor-beta homolog [Mus sp.] |
| T5 | gi|12848236 | unnamed protein product [Mus musculus] |
| T5 | gi|12852148 | unnamed protein product [Mus musculus] |
| T5 | gi|20149756 | eukaryotic initiation factor 4A-III [Mus musculus] |
| T5 | gi|556308 | protein synthesis initiation factor 4A [Mus musculus] |
| T5 | gi|12856949 | unnamed protein product [Mus musculus] |
| T5 | gi|13385872 | interleukin enhancer-binding factor 2 [Mus musculus] |
| T5 | gi|312922382 | aldolase 1 A retrogene 1 [Mus musculus] |
| T5 | gi|26328229 | unnamed protein product [Mus musculus] |
| T5 | gi|33859604 | 26S protease regulatory subunit 7 [Mus musculus] |
| T5 | gi|23271821 | Rfc5 protein [Mus musculus] |
| T6 | gi|149751320 | PREDICTED: tropomyosin alpha-3 chain-like isoform 1 [Equus caballus] |
| T6 | gi|27434482 | ubiquitin ligase E3 alpha-II [Mus musculus] |
| T6 | gi|398050 | ribosomal protein L18 [Mus musculus] |
| T6 | gi|40254525 | tropomyosin alpha-3 chain [Mus musculus] |
| T6 | gi|7305443 | 60S ribosomal protein L7a [Mus musculus] |
| T6 | gi|28077023 | testis-expressed protein 19.1 [Mus musculus] |
| T6 | gi|254675270 | 40S ribosomal protein S5 [Mus musculus] |
| T6 | gi|4506743 | 40S ribosomal protein S8 [Homo sapiens] |
| T6 | gi|148694198 | tropomyosin 1, alpha, isoform CRA_f [Mus musculus] |
| T6 | gi|31560030 | tropomyosin alpha-1 chain isoform 3 [Mus musculus] |
| T6 | gi|12833697 | unnamed protein product [Mus musculus] |
| T6 | gi|4506619 | 60S ribosomal protein L24 [Homo sapiens] |
| T6 | gi|148684442 | ribosomal protein S3, isoform CRA_d [Mus musculus] |
| T6 | gi|6755372 | 40S ribosomal protein S3 [Mus musculus] |
| T6 | gi|148680472 | mCG124430 [Mus musculus] |
| T6 | gi|22094075 | ADP/ATP translocase 2 [Mus musculus] |
| T6 | gi|7021537 | U2 small nuclear ribonucleoprotein A' [Mus musculus] |
| T6 | gi|12833651 | unnamed protein product [Mus musculus] |
| T6 | gi|4506725 | 40S ribosomal protein S4, X isoform X isoform [Homo sapiens] |
| T6 | gi|7638398 | epidermal keratin 10 [Mus musculus] |
| T6 | gi|4506741 | 40S ribosomal protein S7 [Homo sapiens] |
| T6 | gi|2500367 | RecName: Full=60S ribosomal protein L21 |
| T6 | gi|148693404 | mCG14980 [Mus musculus] |
| T7 | gi|17986258 | myosin light polypeptide 6 isoform 1 [Homo sapiens] |
| T7 | gi|71037403 | myosin light chain, regulatory B-like [Mus musculus] |
| T7 | gi|200796 | 16S ribosomal protein [Mus musculus] |
| T7 | gi|293651567 | E3 ubiquitin-protein ligase UBR2 isoform 2 [Mus musculus] |
| T7 | gi|4506631 | 60S ribosomal protein L30 [Homo sapiens] |
| T7 | gi|6755368 | 40S ribosomal protein S18 [Mus musculus] |
| T7 | gi|3097244 | ribosomal protein S14 [Mus musculus] |
| T7 | gi|71664 | calmodulin - salmon |
| T7 | gi|53300 | ventricular alkali myosin light chain [Mus musculus] |
| T7 | gi|26354775 | unnamed protein product [Mus musculus] |
| T7 | gi|148672594 | mCG8804 [Mus musculus] |
| T7 | gi|4506707 | 40S ribosomal protein S25 [Homo sapiens] |
| T7 | gi|50321 | unnamed protein product [Mus musculus] |
| T7 | gi|82890078 | PREDICTED: 60S ribosomal protein L30-like [Mus musculus] |
| T7 | gi|4506623 | 60S ribosomal protein L27 [Homo sapiens] |
| T7 | gi|4506701 | 40S ribosomal protein S23 [Homo sapiens] |
| T7 | gi|74198639 | unnamed protein product [Mus musculus] |
| T7 | gi|12963511 | 40S ribosomal protein S19 [Mus musculus] |
| T7 | gi|4506697 | 40S ribosomal protein S20 isoform 2 [Homo sapiens] |
| T7 | gi|6677775 | 60S ribosomal protein L22 [Mus musculus] |
| T7 | gi|4506681 | 40S ribosomal protein S11 [Homo sapiens] |
| T7 | gi|5542285 | Chain A, Crystal Structure Of Macrophage Migration Inhibitory Factor Complexed With (E)-2-Fluoro-P-Hydroxycinnamate |
| T7 | gi|4506605 | 60S ribosomal protein L23 [Homo sapiens] |
| T7 | gi|4506685 | 40S ribosomal protein S13 [Homo sapiens] |
| T7 | gi|51304 | unnamed protein product [Mus musculus] |
| T7 | gi|14165469 | 40S ribosomal protein S15a [Homo sapiens] |
| T7 | gi|13385044 | 60S ribosomal protein L35 [Mus musculus] |
| T7 | gi|82918395 | PREDICTED: 40S ribosomal protein S13-like [Mus musculus] |
| T7 | gi|4506711 | 40S ribosomal protein S27 [Homo sapiens] |
| T7 | gi|148686760 | mCG13052 [Mus musculus] |
| T7 | gi|6677801 | 40S ribosomal protein S17 [Mus musculus] |
| T7 | gi|6755911 | thioredoxin [Mus musculus] |
| T7 | gi|13385408 | 60S ribosomal protein L11 [Mus musculus] |
| T7 | gi|817939 | histone H2A [Mus musculus domesticus] |
| T7 | gi|4506621 | 60S ribosomal protein L26 [Homo sapiens] |
| T7 | gi|9790041 | ubiquitin-conjugating enzyme E2 A [Mus musculus] |
| T7 | gi|4506703 | 40S ribosomal protein S24 isoform c [Homo sapiens] |
| T7 | gi|149270415 | PREDICTED: 40S ribosomal protein S15a-like [Mus musculus] |
| T7 | gi|55741555 | 60S ribosomal protein L34 isoform 2 [Mus musculus] |
| T7 | gi|13097093 | Krt14 protein [Mus musculus] |
| T7 | gi|9790219 | destrin [Mus musculus] |
| T7 | gi|14277700 | 40S ribosomal protein S12 [Homo sapiens] |
| T7 | gi|387496 | tumor metastatic process-associated protein NM23 [Mus musculus] |
| T7 | gi|6677779 | 60S ribosomal protein L28 [Mus musculus] |
| T7 | gi|6671746 | cofilin-2 [Mus musculus] |
| T7 | gi|4506633 | 60S ribosomal protein L31 isoform 1 [Homo sapiens] |
| T7 | gi|12832665 | unnamed protein product [Mus musculus] |
| T7 | gi|5031595 | actin-related protein 2/3 complex subunit 4 isoform a [Homo sapiens] |
| T7 | gi|6679753 | 40S ribosomal protein S30 precursor [Mus musculus] |
| T7 | gi|148678766 | mCG15017 [Mus musculus] |
| T7 | gi|28077023 | testis-expressed protein 19.1 [Mus musculus] |
| T7 | gi|14589953 | DNA-directed RNA polymerases I, II, and III subunit RPABC3 [Homo sapiens] |
| T7 | gi|7305247 | lysozyme C-1 precursor [Mus musculus] |
| T7 | gi|12963737 | exportin-2 [Mus musculus] |
| T7 | gi|1350733 | RecName: Full=60S ribosomal protein L36 |
| T7 | gi|148686998 | mCG50210 [Mus musculus] |
| T7 | gi|4759160 | small nuclear ribonucleoprotein Sm D3 [Homo sapiens] |
