# Supplementary material for: Mobilization of LINE-1 retrotransposons is restricted by Tex19.1 in mouse embryonic stem cells
Source: eLife. 2017 Aug 14;6:e26152. doi: 10.7554/eLife.26152 (PMC5570191; doi:10.7554/eLife.26152)
Supplement: Supplementary file 2. — Proteins identified by mass spectrometry in TEX19.1-YFP immunoprecipitates from mouse ESC cytoplasmic lysates, but not in YFP controls. Only interactors verified by Western blotting (Figure 2) are listed. Queries matched indicates the number of MS/MS spectra that were matched to each protein, coverage indicates the percentage of target protein matched by MS/MS spectra. DOI: http://dx.doi.org/10.7554/eLife.26152.023 [file elife-26152-supp2.doc]

### Supplementary file 2. Proteins Identified In TEX19.1-YFP Immunoprecipitates.

| **Protein name** | **Gel region (kD)** | **Queries matched** | **Protein Mass (kD)** | **Coverage** | **Description/Reference** |
| --- | --- | --- | --- | --- | --- |
| UBR2 | ~110-260 | 504 | 202.457 | 65.8% | RING domain E3 ubiquitin-protein ligase. N-recognin operating in the N-end rule pathway for protein degradation (Kwon et al. 2003). |
| TEX19.1-YFP fusion | ~55-75 | 60 | 67.374 | 24.4% | Identified in screen for testis-expressed genes (Wang et al. 2001), expressed in germ cells, pluripotent cells and hypomethylated placenta (Hackett et al. 2012; Kuntz et al. 2008; Öllinger et al. 2008; Reichmann et al. 2013). Has roles in spermatogenesis and placenta development, represses retrotransposons in these tissues (Öllinger et al. 2008; Reichmann et al. 2013). |
| HUWE1 | ≥260 | 39 | 486.295 | 10.7% | HECT domain E3 ubiquitin ligase. Multiple substrates reported including histones and p53 (Chen et al. 2005; Liu et al. 2005). |
| UBE2A/B | ≤20 | 2 | 17.419 | 8.5% | E2 ubiquitin-conjugating enzyme. RAD6 homolog, interacts and functions with UBR2 (Kwon et al. 2003; Xie and Varshavsky 1999). |
