# Supplementary material for: Mobilization of LINE-1 retrotransposons is restricted by Tex19.1 in mouse embryonic stem cells
Source: eLife. 2017 Aug 14;6:e26152. doi: 10.7554/eLife.26152 (PMC5570191; doi:10.7554/eLife.26152)
Supplement: Supplementary file 3. — Lower case nucleotides in the repair template sequence indicate mutations relative to wild-type sequence. DOI: http://dx.doi.org/10.7554/eLife.26152.024 [file elife-26152-supp3.doc]

### Supplementary file 3. Oligonucleotides Used In This Study.

| Primer | Sequence | |
| --- | --- | --- |
| mLINE-1 ORF2 qRT-PCR f | 5'-GGAGGGACATTTCATTCTCATC-3' |  |
| mLINE-1 ORF2 qRT-PCR r | 5'-GCTGCTCTTGTATTTGGAGCATAGA-3' |  |
| mLINE-1 A qRT-PCR f | 5'-TCTGGTGAGTGGAACACAGC-3' |  |
| mLINE-1 A qRT-PCR r | 5'-AGTCTCGAGTGGAGCGGAAG-3' |  |
| mLINE-1 Tf qRT-PCR f | 5'-GCCTAAGCCACAGCAGCA-3' |  |
| mLINE-1 Tf qRT-PCR r | 5'-GCTGTCAGGTTCTCTGGCG-3' |  |
| mLINE-1 Gf qRT-PCR f | 5'-TGGAATACAGAGTGCCAGCC-3' |  |
| mLINE-1 Gf qRT-PCR r | 5'-GTGCTCTCACCAGGAAGGTG-3' |  |
| *Tex19.1* qRT-PCR f | 5'-AAAATGGGCCACCCACATCTC-3' |  |
| *Tex19.1* qRT-PCR r | 5'-CCACTGGCCCTTGGACCAGAC-3' |  |
| β-actin Ex2 qRT-PCR f | 5'-GGCTGTATTCCCCTCCATCG-3' |  |
| β-actin Ex3 qRT-PCR r | 5'-ACATGGCATTGTTACCAACTGG-3' |  |
| β-actin Ex4 qRT-PCR f | 5'-ACCGAGCGCGGCTACAG-3' |  |
| β-actin Ex4 qRT-PCR r | 5'-CTTAATGTCACGCACGATTTCC-3' |  |
| ORF1-T7 qRT-PCR f | 5'-TGAGAGATTTTGTCACCACCAG-3' |  |
| ORF1-T7 qRT-PCR r | 5'-TAACCCATTTGCTGTCCACC-3' |  |
| *GAPDH* qRT-PCR f | 5'-CAGCCTCAAGATCATCAGCA-3' |  |
| *GAPDH* qRT-PCR r | 5'-GTCTTCTGGGTGGCAGTGAT-3' |  |
| *Ubr2* CRISPR guide A1 | 5'-AAACGTGTTTTATGCATGGAGTGCC-3' |  |
| *Ubr2* CRISPR guide A2 | 5'-CACCGGCACTCCATGCATAAAACAC-3' |  |
| *Ubr2* CRISPR guide B1 | 5'-AAACATATCGATGGTCTCTATGGAC-3' |  |
| *Ubr2* CRISPR guide B2 | 5'-CACCGTCCATAGAGACCATCGATAT-3' |  |
| *Tex19.1* ES genotype f | 5'-CGCTCAGTCAGGGAGGTAAG-3' |  |
| *Tex19.1* ES genotype r | 5'-GCTGCCTCTTTCCTGTGTTC-3' |  |
| CRISPR T7 guide A PCR | 5'-TGTAATACGACTCACTATAGGGAAACGTGTTTTATGCAT  GGAGTGCC-3' |  |
| CRISPR T7 guide B PCR | 5'-TGTAATACGACTCACTATAGGGAAACATATCGATGGTCT  CTATGGAC-3' |  |
| CRISPR universal guide PCR | 5'-AAAAGCACCGACTCGGTGCC-3' |  |
| *Ubr2* CRISPR repair template | 5'-GACTGTGCAGTTGACCCCAtCTagaTTTTATGCATGGAGT  GCTTCCTGGGAAGTATCCATAGAGACCATCGATATAGG-3' |  |
| Ubr2 genotyping f | 5'-TCTGAGGTTGCAAGAGAATGT-3' |  |
| Ubr2 genotyping r | 5'-GGCCACAGATCAGCTAAACC-3' |  |
