# Supplementary material for: Mobilization of LINE-1 retrotransposons is restricted by Tex19.1 in mouse embryonic stem cells
Source: eLife. 2017 Aug 14;6:e26152. doi: 10.7554/eLife.26152 (PMC5570191; doi:10.7554/eLife.26152)
Supplement: Supplementary file 4. — Description of plasmids used in this study. DOI: http://dx.doi.org/10.7554/eLife.26152.025 [file elife-26152-supp4.doc]

### **Supplementary file 4. Plasmids Used In This Study.**

| **Plasmid** | **Description** |
| --- | --- |
| 99-gfp-LRE3 | Described previously (Coufal et al. 2009), contains a full-length retrotransposition-competent human L1 element (LRE3) (Brouha et al. 2002) tagged with the *mEGFPI* retrotransposition indicator cassette (Ostertag et al. 2000) in pCEP4 (Invitrogen) lacking a CMV promoter and containing a puromycin rather than a hygromycin resistance cassette (Garcia-Perez et al. 2010). |
| 99-gfp-JM111 | Described previously (Coufal et al. 2009). A derivative of 99-gfp-LRE3 where the cloned human L1, L1RP (Kimberland et al. 1999), contains two engineered missense mutations in hL1-ORF1p (R261A, R262A). |
| 99-gfp-TGF21 | Described previously (Garcia-Perez et al. 2010), contains a full-length retrotransposition-competent mouse L1GF element (TGF21) (Goodier et al. 2001) tagged with the *mEGFPI* retrotransposition indicator cassette (Ostertag et al. 2000) cloned in pCEP4 (Invitrogen) lacking a CMV promoter and containing a puromycin rather than a hygromycin resistance cassette (Garcia-Perez et al. 2010). |
| 99-gfp-L1SM | Described previously (Garcia-Perez et al. 2010), contains a full-length retrotransposition-competent codon-optimized mouse L1 T_F_ element (L1spa codon-optimized) (Han and Boeke 2004) tagged with the *mEGFPI* retrotransposition indicator cassette (Ostertag et al. 2000) cloned in pCEP4 (Invitrogen) lacking a CMV promoter and containing a puromycin rather than a hygromycin resistance cassette (Garcia-Perez et al. 2010). |
| 99-gfp-L1SMmut2 | Described previously (Garcia-Perez et al. 2010), a derivative of 99-gfp-L1SM where the cloned mouse L1 contains two missense mutations in the endonuclease and reverse transcriptase domains of ORF2p (D212G and D709Y respectively) (Han and Boeke 2004). |
| pCEPL1SM | Described previously (Han and Boeke 2004), contains a full-length retrotransposition-competent codon-optimized mouse L1 T_F_ element (L1spa codon-optimized) (Han and Boeke 2004) tagged with the *mneoI* retrotransposition indicator cassette (Freeman et al. 1994) and cloned in pCEP4 (Invitrogen). |
| pCEPL1SM-T7 | A derivative of pCEPL1SM where a T7-epitope tag has been cloned in the C-terminus of ORF1p. Also, the L1 element lacks the 5’ UTR from L1spa. |
| pCEPL1SM-T7-ORF1^RA^ | A derivative of pCEPL1SM-T7 that contains two missense mutations in the RNA binding domain of ORF1p (R297A, R298A). These mutations abolish the ability of the codon-optimized L1 T_F_ element to mobilize (Figure 2 – figure supplement 1F). |
| pADE2TE1 | Described previously (Doucet et al. 2010), contains a full-length retrotransposition-competent human L1 (L1.3) (Sassaman et al. 1997) tagged with the *mneoI* retrotransposition indicator cassette (Freeman et al. 1994) and cloned pCEP4 (Invitrogen). A T7 and a TAP epitope tag have been cloned in the C-terminus of ORF1p and ORF2p respectively. |
| pCEPL1SMN21A | Described previously (Alisch et al. 2006), a derivative of pCEPL1SM that contains a missense mutation in the endonuclease domain of ORF2p (N21A). |
| Zfl2-2mneoI | Described previously (Sugano et al. 2006), contains a full-length retrotransposition-competent zebrafish LINE-2 element tagged with the *mneoI* retrotransposition indicator cassette (Freeman et al. 1994) and cloned in pCEP4 (Invitrogen). |
| pU6ineo | Described previously (Richardson et al. 2014b), contains the neomycin phosphotransferase expression cassette from pEGFP-N1 (Clontech) cloned in a pBluescript II KS (pBSKS, Stratagene). |
| JJ101/L1.3 | Described previously (Beck et al. 2010), contains a full-length retrotransposition-competent human L1 (L1.3) (Sassaman et al. 1997) tagged with the *mblastI* retrotransposition indicator cassette (Goodier et al. 2007) and cloned in pCEP4 (Invitrogen). |
| JJL1SM | Contains a full-length retrotransposition-competent mouse L1 T_F_ element (L1spa codon-optimized) (Han and Boeke 2004) tagged with the *mblastI* retrotransposition indicator cassette (Goodier et al. 2007) and cloned in pCEP4 (Invitrogen). |
| pCMV5-hORF1-T7 | Described previously (Kulpa and Moran 2005), contains the coding sequence of L1.3 hL1-ORF1p (Sassaman et al. 1997) tagged with a C-terminal T7 epitope in vector pCMV5. |
| pCMV5-hORF1-RA-T7 | A derivative of pCMV5-hORF1-T7 where the coding sequence of ORF1p contains two missense mutation in the RNA binding domain (R261A, R262A). |
| pCMV5-mORF1-T7 | Contains the coding sequence of ORF1p from the codon-optimized mouse L1 T_F_ element (Han and Boeke 2004) tagged with a C-terminal T7 epitope in vector pCMV5. |
| pCMV5-mORF1- RA-T7 | A derivative of pCMV5-mORF1-T7 where the coding sequence of ORF1p contains two missense mutation in the RNA binding domain (R297A, R298A). |
| pCMV5-hORF1-mCherry | Contains the coding sequence of L1.3 hL1-ORF1p (Sassaman et al. 1997) with a C-terminal mCherry tag (Shaner et al. 2005) in vector pCMV5. |
| pCMV5-mORF1-mCherry | Contains the coding sequence of codon-optimized mouse L1 T_F_ element (Han and Boeke 2004) with a C-terminal mCherry tag (Shaner et al. 2005) in vector pCMV5. |
| pK-APOBEC3A | Described previously (Bogerd et al. 2006a), expression vector for APOBEC3A. |
| pK-β-ARRESTIN | Described previously (Bogerd et al. 2006a), expression vector for β-ARRESTIN. |
| pGL3SV40LUC | Obtained from Promega, luciferase under control of a SV40 promoter. |
| pTKLUC | Obtained from Promega, luciferase under control of a HSV-TK promoter**.** |
| pL1.3FF | Described previously (Heras et al. 2013), contains 5´UTR from a human L1.3 L1 element (Sassaman et al. 1997) cloned in pGL3-Basic luciferase reporter vector (Promega). |
| pL1spaFF | Described previously (Heras et al. 2013), contains 5’UTR from a mouse L1 T_F_ element (L1spa) cloned into pGL3-Basic (Promega) |
| pCEP-EGFP | Described previously (Alisch et al. 2006), has the enhanced green fluorescent protein (EGFP) coding sequence in pCEP4 (Invitrogen). |
| pcDNA6.1/mycA | Obtained from Invitrogen. contains a blastidicin resistance cassette. |
| pBSKS | Obtained from Stratagene, pBluescript II KS phagemid cloning vector. |
| pCAG-YFP | The CMV promoter in pEYFP-N1 (Clontech) was replaced with the CAG promoter from pCAGGS (Niwa et al. 1991). |
| pCAG-Tex19.1-YFP | The mouse TEX19.1 open reading frame was inserted into the pCAG-YFP plasmid to tag TEX19.1 on the C-terminus with EYFP. |
| pCAG-TEX19-YFP | The human TEX19 open reading frame was inserted into the pCAG-YFP plasmid to tag TEX19 on the C-terminus with EYFP. |
| pCMV-TEX19 | Human TEX19 under control of a CMV promoter in pCMV-SPORT6 |
| p105 | Obtained from IBA Life Sciences, pEXPR-IBA105 eukaryotic expression vector with CMV promoter and Strep-Tactin (Strep) tag. |
| p105-Tex19.1 | Mouse *Tex19.1* coding sequence cloned into p105 to generate a Strep tag at the N-terminus. |
| p105-TEX19 | Human *TEX19* coding sequence cloned into p105 to generate a Strep tag at the N-terminus. |
| p3xFLAG-CMV-Ubr2 | Mouse *Ubr2* coding sequence cloned in to p3XFLAG-CMV-10 (Sigma) expression vector that generate a 3xFLAG epitope tag at the N-terminus of UBR2. |
| pEGFP3N1-Ubr2 | Mouse *Ubr2* coding sequence cloned into pEGFP-N1 (Clontech) expression vector to generate a GFP tag at the C-terminus of UBR2. |
| pCMV-His_6_-myc-ubiquitin | Described previously (Ward et al. 1995), vector expressing ubiquitin tagged with hexahistidine and myc epitopes. |
| pRF | Described previously (Li et al. 2006), dicistronic reporter construct containing Renilla and firefly luciferase coding sequences. |
| pRFA | Described previously (Li et al. 2006), 400 bp upstream of mL1-ORF1p (L1spa 5' UTR) inserted between luciferase coding sequences in pRF. |
| pRFD | Described previously (Li et al. 2006), 200 bp upstream of mL1-ORF2p (L1spa intergenic region) inserted between luciferase coding sequences in pRF. |
| pRF3 | Described previously (Li et al. 2006), 312 bp from the 3'UTR of mouse L1 (L1spa 3'UTR) inserted between luciferase coding sequences in pRF. |
| pnEA-pS-hL1ORF1p (AII) | hL1-ORF1p coding sequence from pJM101 (Moran et al. 1996) inserted into pnEA-pS (Diebold et al. 2011) for bacterial expression and to generate a cleavable Strep tag at the N-terminus (engineered point mutations: M121A, M125I, M128I) (Khazina et al. 2011). |
| pnEA-pS-hL1ORF1p (AII)-1-152 | A fragment of human LINE-1 ORF1 coding sequence from pJM101 (Moran et al. 1996) inserted into pnEA-pS (Diebold et al. 2011) for bacterial expression and to generate a cleavable STREP tag at the N-terminus (Engineered point mutations: M121A, M125I, M128I) (Khazina et al. 2011). |
| pnEA-pS-hL1ORF1p (AII)-104-338 | A fragment of human LINE-1 ORF1 coding sequence from pJM101 (Moran et al. 1996) inserted into pnEA-pS (Diebold et al. 2011) for bacterial expression and to generate a cleavable STREP tag at the N-terminus (Engineered point mutations: M121A, M125I, M128I) (Khazina et al. 2011). |
| pnEA-pS-B | GB1 coding sequence (Cheng and Patel 2004) inserted into pnEA-pS (Diebold et al. 2011) for bacterial expression and to generate a Strep tag at the N-terminus. |
| pnEA-pS-G | GST coding sequence inserted into pnEA-pS (Diebold et al. 2011) for bacterial expression and to generate a STREP tag at the N-terminus. |
| pnYC-pM-hTex19-pBH | Human *TEX19* coding sequence from pCMV-TEX19 inserted into pnYC-pM (Diebold et al. 2011) for bacterial expression and to generate a cleavable MBP tag at the N-terminus. A cleavable C-terminal GB1 tag (Cheng and Patel 2004) was also introduced, followed by a hexahistidine tag. |
| pnYC-pM-hTex19-1-68-pBH | A fragment of human *TEX19* coding sequence from pCMV14-Tex19 inserted into pnYC-pM (Diebold et al. 2011) for bacterial expression and to generate a cleavable MBP tag at the N-terminus. Furthermore, a cleavable C-terminal GB1 tag (Cheng and Patel 2004) was introduced, followed by a hexa-histidine tag. |
| pnYC-pM-hTex19-69-164-pBH | A fragment of human *TEX19* coding sequence from pCMV14-Tex19 inserted into pnYC-pM (Diebold et al. 2011) for bacterial expression and to generate a cleavable MBP tag at the N-terminus. Furthermore, a cleavable C-terminal GB1 tag (Cheng and Patel 2004) was introduced, followed by a hexa-histidine tag. |
