# Supplementary material for: Mobilization of LINE-1 retrotransposons is restricted by Tex19.1 in mouse embryonic stem cells
Source: eLife. 2017 Aug 14;6:e26152. doi: 10.7554/eLife.26152 (PMC5570191; doi:10.7554/eLife.26152)
Supplement: Supplementary file 5. — List of antibodies, sources and dilutions used for Western blots. DOI: http://dx.doi.org/10.7554/eLife.26152.026 [file elife-26152-supp5.doc]

### **Supplementary file 5. Antibodies Used For Western Blots.**

| **Antibody** | **Source** | **Dilution** |
| --- | --- | --- |
| Rabbit anti-TEX19.1 | Ian Adams (Öllinger et al. 2008) | 1:100 |
| Rabbit anti-mouse L1-ORF1p | Sandy Martin/Alex Bortvin (Martin and Branciforte 1993; Soper et al. 2008) | 1:2000 |
| Rabbit anti-human L1-ORF1p | Oliver Weichenrieder | 1:2000 |
| Mouse anti-β-ACTIN | Sigma-Aldrich Cat# A5441 RRID:AB_476744 | 1:5000 |
| Mouse anti-UBR2 | Abcam Cat# ab57407 RRID:AB_2210171 | 1:1000 |
| Goat anti-UBR2 | Aviva Systems Biology Cat# OAEB00482 RRID:AB_10876360 | 1:100 |
| Rabbit anti-PABP1 | Niki Gray (Burgess et al. 2011) | 1:10000 |
| Rabbit anti-HUWE1 | Bethyl Cat# A300-486A RRID:AB_2264590 | 1:500 |
| Rabbit anti-UBE2A | GeneTex Cat# GTX100426 RRID:AB_1241237 | 1:3000 |
| Mouse anti-GFP/YFP | Roche Cat# 11814460001 RRID:AB_390913 | 1:2000 |
| Mouse anti-RFP/mCherry | ChromoTek Cat# 6g6-100 RRID:AB_2631395 | 1:5000 |
| Rabbit anti-T7 | Abcam Cat# ab18611 RRID:AB_444563 | 1:5000 |
| Rabbit anti-Lamin B1 | Abcam Cat# ab16048 RRID:AB_10107828 | 1:5000 |
| Mouse anti-c-Myc | Sigma-Aldrich Cat# M4439 RRID:AB_439694 | 1:5000 |
| Rabbit anti-TEX19 | Abcam (ab185507) | 1:2500 |
| Rabbit anti-histone H3 | Abcam Cat# ab1791 RRID:AB_302613 | 1:20000 |
| Rabbit anti-p53 | Santa Cruz Biotechnology Cat# sc-6243 RRID:AB_653753 | 1:300 |
